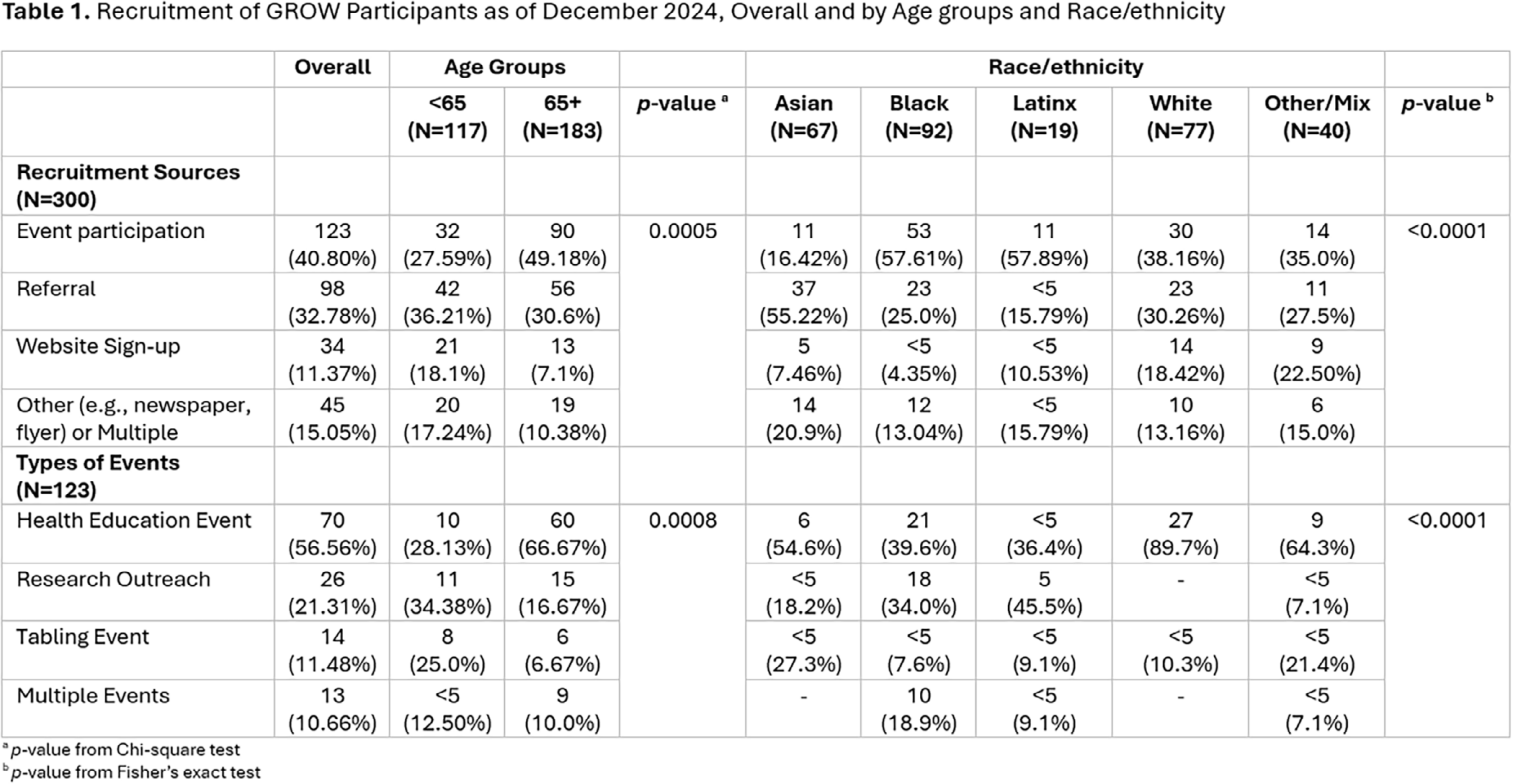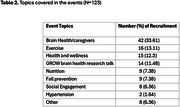# Growing a Diverse Community‐Based Cohort of Women: Lessons Learned from Recruitment Methods in the Greater Sacramento Women's Brain Health (GROW) Study

**DOI:** 10.1002/alz70860_107446

**Published:** 2025-12-23

**Authors:** Rifat B. Alam, Oanh L. Meyer, Kristen M. George, Anna Garzon, Casey T. Castro, Magaly Quinteros, Alexander Ivan B. Posis, Rachel A. Whitmer

**Affiliations:** ^1^ University of California, Davis, Davis, CA, USA; ^2^ University of California, Davis School of Medicine, Sacramento, CA, USA

## Abstract

**Background:**

Studies of lifecourse brain health in diverse women are limited. Community‐based participation and tailored recruitment methods may enhance inclusivity. We describe and evaluate recruitment methods and participation in the GROW Study (2023‐present).

**Method:**

GROW is an observational cohort study of women aged 45+ from the greater Sacramento area in California. GROW investigates risk and resilience factors for cognitive impairment and dementia via questionnaires, annual physical exams, neuropsychological testing, blood draws, and neuroimaging. Recruitment began in December 2023 following community health events that started in October 2022, with the goal of recruiting 700 participants by March 2026. Additional recruitment sources included referrals (word‐of‐mouth from GROW participants or others, referrals from community advisory board members), sign‐ups via the study website, and other methods (e.g., newspaper, flyer). We describe recruitment sources, types of health events, and how success in reaching the target enrollment differed by demographic factors.

**Result:**

As of December 2024, GROW has enrolled 300 participants (mean age=66.9±9.7; 22.3% Asian, 30.7% Black, 6.3% Latinx, 13.3% Multiracial/Other, 25.7% White). 37% of participants have less than a college education. Overall, 40.8% were recruited through events (Table 1). Among those aged 65+, 49.2% were recruited via events, compared to 28.2% of those under 65. Black (57.6%) and Latinx (57.9%) participants were more likely to be recruited via events. Most participants who were referral‐recruited were <65 years old (36.8%) and Asian (55.2%). Black and Latinx participants engaged in both health education events (39.6% and 36.4%, respectively) and research outreach focusing research talks (34.0% and 45.5%, respectively), while Asian, White and Other/Multiracial participants predominantly attended health education programs (54.6%, 89.7% and 64.3%, respectively). A range of topics were covered during events including brain health, caregiving, exercise, and health and wellness (Table 2).

**Conclusion:**

The GROW study successfully enrolled 300 racially and ethnically as well as socioeconomically diverse women over a year. Recruitment sources varied across groups reflecting community engagement being more effective for some, and relationships and trust‐building for others. Our findings highlight that tailored strategies, such as fostering community partnerships and trust, enhance recruitment effectiveness and improve the representation of a diverse population.